# ‘Don’t show that you’re scared’: resilience in providing healthcare in a UK low-to-medium secure hospital

**DOI:** 10.1080/21642850.2021.1874956

**Published:** 2021-01-28

**Authors:** Margaret Husted, Rheyanne Dalton

**Affiliations:** Psychology Department, University of Winchester, Winchester, UK

**Keywords:** Burnout, resilience, healthcare nursing, correctional staff, thematic analysis

## Abstract

**Background:** Healthcare provision within specialist hospitals is associated with heightened levels of stress and burnout, risking negative implications for employees, organisations, and patients. Minimal research has focused on lower-skilled worker experiences. This study explores frontline care workers’ experience and perceptions of providing care within a low-to-medium secure hospital within the UK.

**Method:** Eight semi-structured interviews were conducted with healthcare assistants and mental health nurses (18–65 years) at a low-to-medium secure hospital. Thematic analysis (Braun & Clarke, 2006) was used to analyse the data.

**Results:** Three main themes are proposed: ‘Resilience to threat’ capturing the daily occurrence and normalisation of threat; ‘Need for support’ shows peer to peer talk as the primary coping mechanism but importantly, a possible disconnect between perceptions, and provision, of organisational support; finally, ‘Unique environment’ highlights the dual aspect of providing care and correction whilst coping with common challenges e.g. staff shortages.

**Conclusions:** Results provide insight into the pressures experienced by frontline healthcare workers alongside staff motivation to ‘make a difference’. Findings highlight some unique challenges of working in low-to-medium secure hospitals which contributes to negative outcomes for worker engagement, performance, and individuals’ mental and physical health. Implications for organisations and future practice are discussed.

## Introduction

Human service work is important within the society responsible for providing essential and emergency care to those in need (Allen & Palk, 2018). Care is often required among highly complex or chaotic circumstances, which increases the challenge of delivering intended services (Shakespeare-Finch & Daley, 2017). Employees are ultimately responsible for improving and maintaining the physical and psychological well-being of others, often in situations where there is minimal room for a mistake (Dollard, Dormann, Boyd, Winefield, & Winefield, 2003). Incorrect care can result in life-changing or life-ending consequences (Dollard et al., 2003). However, it is suggested for one to sufficiently care for someone else, one must be sufficiently cared for oneself (Silva et al., 2009) bringing the importance of promoting, and maintaining, the physical and psychological well-being of human service workers to the forefront.

However, high levels of stress are commonly reported amongst employees within the sector (Johnson et al., 2005; Oginska-Bulik, 2005). Heightened or prolonged levels of stress can result in numerous negative ramifications; causing harm to employees, organisations, service users, and associated friends and families (Griffin, Hogan, Lambert, Tucker-Gail, & Baker, 2010; Johnson et al., 2005). Workplace stress is detrimental to employees’ physical and psychological health; reducing the overall quality of life (Berg, Hem, Lau, & Ekeberg, 2006; Gould, Watson, Price, & Valliant, 2013). Furthermore, workplace stress can in severe cases result in burnout, a form of psychological exhaustion (Khamisa, Oldenburg, Peltzer, & Ilic, 2015) whereby an individual is suggested to experience emotional exhaustion, depersonalisation, and a reduced sense of personal accomplishment (Gould et al., 2013; Griffin et al., 2010)**.**

Employees experiencing workplace stress or burnout are more likely to demonstrate unhealthy behaviours such as rumination, lack of sleep, and substance misuse (Allen & Palk, 2018). Such behaviours along with levels of stress or burnout are predictive of poor health and staff absenteeism (Allen & Palk, 2018; Griffin et al., 2010). Further, they increase the risk of individuals leaving the profession (Koen, Van Eeden, & Wissing, 2011) which impacts employees who remain at work by further reducing staff resources, skill, and support (Lambert, Edwards, Camp, & Saylor, 2005) and is costly for organisations through expenditure on sick pay, new staff recruitment, and training (Lambert et al., 2005). Maintaining good levels of trust and rapport between service providers and service users is fundamental to providing high-quality care (Mor Barak, Nissly, & Levin, 2001). However, staff absenteeism and turnover are detrimental to service provider-user relationships (Mor Barak et al., 2001). Evidence indicates some employees remain at work despite experiencing burnout (Khamisa et al., 2015) which negatively impacts on care quality, attitudes and performance at work (Stewart & Terry, 2014), seen by increased impersonal patient interactions and disengagement with job roles (Griffin et al., 2010).

Human service workers experience a magnitude of workplace stressors, ranging from minor, persistent stressors to major, serious events (Lambert, Lambert, Petrini, Li, & Zhang, 2007). One predictor of workplace stress is role problems, with issues surrounding workload, role ambiguity, and role conflict (Lambert, Hogan, & Tucker, 2009). Regarding workload, employees are required to complete numerous tasks simultaneously; attending to the individual needs of multiple service users (Ducharme, Knudsen, & Roman, 2007; Pillay, 2009). Adding to this challenge is a lack of time, staff, and resources due to financial cuts (Griffin et al., 2010). Role ambiguity occurs where ill-defined job roles cause confusion, crossovers between responsibilities, and increase workload (Lambert et al., 2009). For example, higher-qualified employees may delegate responsibilities to lower-qualified employees who lack adequate training for the task (Zimmermann, 2000). Role conflict is evident when individuals’ feel torn between different responsibilities (Armstrong & Griffin, 2004). For example, prison staffs are often conflicted between roles of care and correction (Gallavan & Newman, 2013). Consequently, multiple role problems increase the challenges of delivering high-standard services.

Another predictor of workplace stress is the social climate (Lambert et al., 2007). This includes concepts of workplace belongingness; including levels of acceptance, respect, and value individuals feel within organisations (Cockshaw & Shochet, 2010). Employees reporting higher levels of social support (especially co-worker support) appear better able to contend with workplace stressors (Gould et al., 2013; Shakespeare-Finch & Daley, 2017). Those reporting a lack of social support in turn are more likely to experience psychological strain and demonstrate intentions to quit (Ducharme et al., 2007).

An important precursor of workplace stress manifests in the traumatic, potentially dangerous, emotional nature of human service work (Lambert et al., 2007; Shakespeare-Finch & Daley, 2017). Employees often work closely with vulnerable individuals at great responsibility and risk, including exposure to trauma and difficult service user behaviour (Dollard et al., 2003; Hogh, Sharipova, & Borg, 2008). Patient self-harm, suicidal attempts, and suicide characterise particularly traumatic events (Holmes & Maclnnes, 2003). This is especially distressing for those employees responsible for preventing such occurrences of trauma (Regehr, Goldberg, & Hughes, 2002; Shakespeare-Finch & Daley, 2017) and increased risk of stress is apparent where employees becoming emotionally invested or empathise with patient’s situations (Allen & Palk, 2018; Johnson et al., 2005).

Although prevalent across most human service occupations, it is suggested individuals working in correctional facilities are at elevated risk of workplace stress and burnout (Gallavan & Newman, 2013). This may be due to the uniqueness of institutions in terms of purpose and physical environment (Armstrong & Griffin, 2004). Correctional employees are tasked with unique challenges of supervising and rehabilitating increasingly unwilling and dangerous individuals (Griffin et al., 2010). Prior research with correctional staff shows that both witnessing violence as well as being victimised increases burnout and impacts on individual’s sense of security (Isenhardt & Hostettler, 2020) with indications of a relationship between inmates-to-staff violence, post-traumatic stress disorder and burnout (Boudoukha, Altintas, Rusinek, Fantini-Hauwel, & Hautekeete, 2013). Further, threats of this nature are also experienced from service user’s friends and families, and occasionally colleagues (Hogh et al., 2008; Pillay, 2009). Employees at particular risk of physical or verbal abuse when delivering sensitive information about a patient’s condition (Allen & Palk, 2018; Pillay, 2009). Yet, human service roles require employees to suppress negative emotions and appear controlled when dealing with difficult service user’s behaviour (Johnson et al., 2005; Oginska-Bulik, 2005). Thus, increasing the risk of emotional dissonance and stress whereby individuals display emotions inconsistent with genuinely felt emotions (Dollard et al., 2003).

The challenges of providing care within correctional facilities are mirrored in low-to-medium secure hospitals. Low-to-medium secure hospitals provide inpatient care and treatment for individuals with complex mental health problems who are a risk to others (or themselves) and need physical security to prevent their escaping. Patients will either have been charged, or convicted, of a criminal offence. Individuals may move between high-security services, such as Ashworth or Broadmoor Hospital, to medium or low secure services e.g. Fairfield Hospital, Wakefield. Changes between service provisions may reflect a positive response to treatment and a reduction in risk, whereas an escalation in risk may require patients to move from low/medium secure to high-security services. Therefore, secure hospitals create a unique challenge of providing treatment but with the additional correctional requirements of constraining patients’ freedom of movement. What is unclear is how this duality may influence health care personal working within such environments. Despite being undervalued by many in the public (Griffin et al., 2010), such institutions provide vital services; therefore, elevated risks of burnout highlight a need to conduct research within this field of work (Gallavan & Newman, 2013).

What has been shown is that the high demand and responsibility of such work requires elements of resiliency amongst several challenging factors (Shakespeare-Finch & Daley, 2017). Resiliency refers to an individual’s ability to ‘bounce back’ when faced with highly challenging or disruptive events, maintaining psychological equilibrium (Bonanno, 2004). As expected, levels of resilience amongst human service workers vary, so where some may thrive, others struggle and leave the profession (Koen et al., 2011). Currently, exits from this field of work outweigh entries; resulting in staff shortages (Koen et al., 2011). This suggests difficulties in attracting and retaining well-trained, human service workers are increasing (Armstrong & Griffin, 2004). Individuals who remain should be highly valued for providing effective service and demonstrating resiliency (Allen & Palk, 2018) but it is also important to identify what helps individuals survive and indeed thrive when providing a human service.

There are indications that extrinsic job satisfaction is relevant to understanding the impact of physical and non-physical violence on nursing staff (Galián-Muñoz, Ruiz-Hernandez, Llor-Esteban, & Lopez-Garcia, 2016). However, although potentially protective in relation to certain aspects, such as levels of cynicism or emotional exhaustion, job satisfaction is not shown to be related to levels of burnout. It is important to also note that much existing literature focuses on higher-skilled professionals; often ignoring ‘lower-skilled’, frontline employees even though without these employees’ unsustainable gaps would occur in healthcare systems (Gould et al., 2013; Oginska-Bulik, 2005; Pillay, 2009). Research that is available suggests such individuals experience similar levels of workplace stress (Armstrong & Griffin, 2004). Consequently, there is a clear need to further explore the causes and consequences of workplace stress amongst alternative, lower-skilled populations. Additionally, much research focuses on quantitative measures of workplace stress (Gallavan & Newman, 2013; Hogh et al., 2008) but there is a need to qualitatively explore employee’s perceptions of providing human services in order to gain an in-depth understanding of what it is like working within fields of care and correction (Koen et al., 2011).

In summary, high levels of workplace stress and burnout amongst human service workers are concerning; suggesting challenging working conditions are causing this work to become unsustainable and less effective in its aims (Armstrong & Griffin, 2004). This is problematic as society relies on care and correction systems that can retain effective staff (Shakespeare-Finch & Daley, 2017). Levels of workplace stress and burnout suggest more needs to be done to help frontline employee’s abilities to be resilient’ (Pillay, 2009). There is a clear need to explore human service workers’ experiences, but more importantly, understand how individuals cope with workplace stressors; something rarely accounted for within literature (Griffin et al., 2010). A greater understanding of how individuals manage stressors could offer insight into inconsistencies of resiliency amongst employees; potentially revealing strategies which could reduce levels of workplace stress and burnout.

### Present study

Based on findings and suggestions presented within the literature, the current study qualitatively explores the experiences, perceptions, and consequences of providing frontline care within a low–medium secure hospital. Focusing on a lower-skilled population which encompass elements of both care and correction, the research aims to explore the apparent influential factors, as well as the coping mechanisms, surrounding workplace stress as perceived by the workers. Specifically, the following research question will be addressed: What are the UK frontline care workers’ experiences and perceptions about providing care within a low–medium secure hospital environment? An aim is for the study findings to have relevance for future practice by providing insight into employee’s needs and therefore informing workplace interventions to promote resilience and well-being amongst frontline care workers within secure settings.

## Method

### Participants and recruitment

This study was conducted using a purposive sample of eight participants: six health care assistants (HCAs) and two registered mental health nurses (RMNs) responsible for the treatment and care of patients within the inpatient facility. Participants comprised six females and two males; with a mean age of 30 years (range: 18–65 years; excluding one participant who did not provide this information) (see Table 1). This sample is appropriate to provide the depth of case-orientated analysis that is fundamental to quality health research and that reflects this specific employment role and context (Vasileiou, Barnett, Thorpe, & Young, 2018).

**Table 1.** Demographic information of study participants.

Participant	00FM	69BN	34WC	33RW	38RH	79RS	92ZB	39ED
Occupation	HCA	HCA	HCA	HCA	HCA	HCA	RMN	RMN
Age (years)	18	22	24	25	25	31	–	65
Gender	Male	Female	Female	Female	Female	Female	Female	Male
Education level	U/G	NVQ	MSC	U/G	A-level	U/G	MSC	U/G

Note: Occupation: HCA, Health Care Assistant; RMN, Registered Mental Health Nurse; Education Level: U/G, Undergraduate degree; NVQ, National Vocational Qualification; MSC, Master degree; A-level, Advanced level qualification.

Participants were recruited via gatekeeper access from a 119 bed, low–medium secure hospital: offering patient-centred, recovery-focused services to adult males. Patients within the facility have complex mental health needs e.g. personality disorder and a history of offending. Security levels range across and between wings of the hospital; from the psychiatric intensive care unit through to rehabilitation flats. This low and medium secure hospital provides patients with an individualised care pathway led by a multidisciplinary team. The model of care followed comprises a mental health pathway, assisting patients from acute medium security through to discharge. Services offered include a range of psychotherapies, behavioural therapies, and activity engagement to optimise mental health recovery and relapse prevention such as substance abuse treatment.

This study was advertised via emails and flyers within the hospital with formal recruitment conducted onsite with the researcher granted temporary access to the hospital for recruitment and data collection.

### Data collection

An initial pilot interview was conducted with a nursing staff member (lasting 40:33 min) to ensure the interview guide was effective in obtaining relevant information. Following piloting, semi-structured, one-to-one interviews were conducted at the low-to-medium secure hospital. All participants received an information sheet at least 24 h prior to participation; including relevant information about the study. Before obtaining written informed consent, participants received another copy of the information sheet and had the opportunity to ask questions. Participants completed a basic demographics form and created a unique four-digit participant number which was used to provide anonymity and to allow for possible participant withdrawal of data/participation – the four-digit codes are used within the paper in place of participants’ real initials etc. Interviews were conducted using a semi-structured interview guide comprising open questions and prompts (see Appendix). Participants were requested to answer questions such as ‘What motivates you to do your job?’ as accurately and honestly as possible. Interviews were audio-recorded for transcribing purposes and lasted between 15:14 and 41:44 min (mean: 25:51 min). Note: the shortest interview ended early as the participant was urgently needed on their ward. Once interviews were complete, participants were debriefed. The debrief included referral information informing participants on how to seek psychological support.

### Data analysis

**Figure 1.**
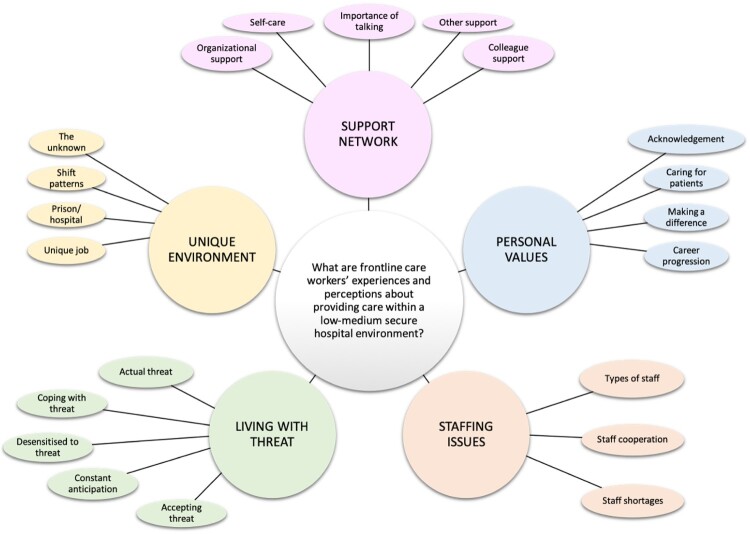
Initial thematic map.

Data were analysed following Braun and Clarke’s (2006) six stages of thematic analysis (familiarise yourself with data; generalise initial codes; search for themes; review themes; define and name themes; produce report). Interviews were transcribed by participant numbers (ensuring anonymity) and re-read several times; allowing the researcher to become familiar with data. All transcripts were then coded and presented in a coding table identifying line numbers of corresponding extracts from transcripts. Codes were colour coded and grouped to assist the search for themes; corresponding extracts were cross-referenced to ensure codes were representative of proposed themes. Initial searches identified five preliminary themes (comprising three–five sub-themes) – see initial thematic map (Figure 1).

**Figure 2.**
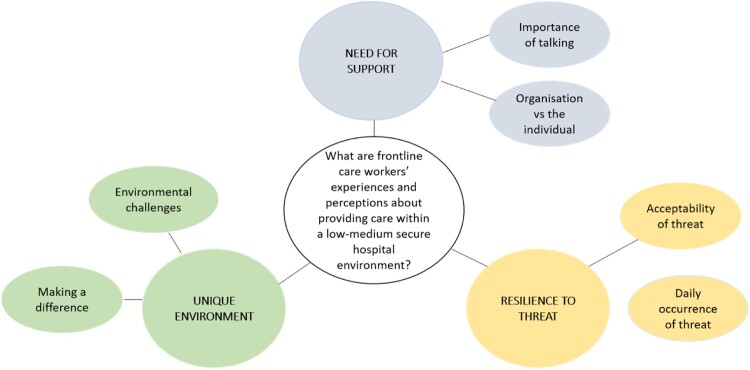
Final thematic map.

Preliminary themes and sub-themes were reviewed, re-organised, and reinterpreted by the research team with regards to the data and research question being considered. Following a final review of the data and interpretative analysis by the research team three main themes are proposed: ‘Living with threat’; ‘Need for support’; ‘Unique environment’ – see Figure 2. To ensure quality of the analysis process, the research team discussed each stage; ensuring codes, themes, and supporting extracts were representative of collected data.

### Reflection

It is important to acknowledge the researcher’s active role throughout this research process; from creating a research question to analysis and write up. It is the researcher’s initial interest and research into the topic of human service work that characterised the current study. Therefore, it is possible that insight into this field of work, via research, may have influenced certain aspects of this study. For example, the majority of existing literature sheds negative light on the impact human service work can have on employees; therefore, pre-existing knowledge of negative consequences could have biased data collection (through creation of the interview guide and subsequent analysis). To prevent bias from occurring, the researcher remained open-minded to possibilities of positive and negative responses from participants regarding perceptions and experiences of providing frontline care. The researcher also practiced and applied providing neutral, non-influencing responses during interview periods. Additionally, throughout analysis, the researcher remained conscious of their own preconceptions to maintain a bracketed approach, paying equal attention to all relevant codes and themes.

### Evaluative criteria

The quality of any psychological study is essential; whereby evaluative criteria ensure research is appropriately conducted (Yardley, 2008). Yardley’s (2000) evaluative criteria of qualitative methodology was adopted for the current study. This criterion is tailored to health research and has been applied to previously validated studies (e.g. Huggett et al., 2018; Künzler-Heule, Beckmann, Mahrer-Imhof, Semela, & Händler-Schuster, 2016; Robinson, Clare, & Evans, 2005). Yardley (2000) proposes four principles to assess the quality of qualitative research: sensitivity to context; commitment and rigour; transparency and coherence; and finally, impact and importance. Researchers suggest this study is evaluated within this context.

## Results and discussion

Due to the qualitative nature of this study discussion of results will be presented alongside findings. When exploring participants’ experiences and perceptions of providing frontline care within a low–medium secure hospital, as shown in Figure 2, the final analysis proposed three main themes: Resilience to threat; Need for support; and Unique environment (each comprising two sub-themes). Themes highlight the prevalence and impact of threat, the perceived need and experiences of support, and the overall uniqueness of the given job.

### Resilience to threat

The first theme ‘Resilience to threat’ captures participants’ experiences and perceptions of being confronted with a threat at work; characterised by violent and aggressive behaviours (physical and verbal) and threats of this kind and how individuals differ in their resilience to this stressor.

#### Daily occurrence of threat

This sub-theme highlights participants’ daily experiences of threat; whereby they discussed regularly receiving or witnessing threats from patients. ‘This is a daily trouble we’ll have patients kicking off … escalating shouting maybe punching’ (33RW: Female, HCA). This suggests threat is a persistent challenge faced by frontline care workers within a low–medium secure environment. Such data is coherent within existing literature suggesting human service workers risk receiving violence and aggression (Rasmussen, Hogh, & Andersen, 2013). Moreover, when questioned how experiences of threat affected them, several participants discussed negative emotional responses ‘nervous … bit anxious’ (33RW: Female, HCA) or an inability to cope ‘the fear kinda spreads amongst people … people started cancelling their shifts there was staff being like “no I’m not coming in”’ (34WC: Female, HCA); ‘there was one weekend that patient yelled at me every day all day for two days … I just couldn’t do it anymore I thought I’m gonna have to quit my job’ (79RS: Female, HCA). These responses are concerning; suggesting participants are negatively affected by and are struggling to cope with the threat. Experiences of cancelling shifts and thoughts of quitting suggest a lack of emotional resilience remaining amongst participants; demonstrating inabilities to ‘bounce back’ from disruptive events (Bonanno, 2004). This suggests that threat is not only a daily occurring issue when providing frontline care, but experiences of threat entail risks of psychological distress, staff absenteeism, and turnover. This mirrors similar issues evident amongst alternative human service populations which have been shown to predict several negative implications (Hogh et al., 2008).

Interestingly, differences occurred when participants discussed how they cope with the threat. Some discussed the need to prevent patient escalation by appearing calm: ‘Don’t show that you’re scared … I can be looking really as calm as hell and inside you’re like a duck that’s paddling away under water’ (39ED: Male, RMN). This suggests some participants engage in emotional dissonance to protect themselves from the risks of threat (Johnson et al., 2005). Despite being discussed as a protective factor, this risks negative implication; whereby repetitive engagement in emotional dissonance is associated with increased stress and reduced psychological well-being (Dollard et al., 2003). Alternatively, some discussed ignoring verbal threats, to remain prepared for physical threat whilst also providing intended services; ‘I’ve had threats patients have threatened to rape me come find my family and hurt them … sometimes you switch off … you don’t listen … you watch their body language’ (79RS: Female, HCA). The ability to ‘switch off’ from certain aspects of threat suggests in some individuals there was a degree of resilience and coping amongst challenging situations (Koen et al., 2011). However, other participants opted instead for avoidance, ‘I cancelled the shift I was on the next day’ (38RH: Female, HCA) confirming risks of absenteeism due to threat. Moreover, avoidance of work may suggest a lack of resilience indicating some individuals struggle or require longer to recover from the threat. This reflects avoidance coping which is suggested to have a negative impact on mental health and increase the difficulty of refacing a given issue (Lambert et al., 2007). Consequently, this sub-theme reveals the daily occurrence of threat when providing frontline care within a low–medium secure hospital, but also suggests inconsistencies in how participants are affected/cope with the threat.

#### Acceptability of threat

The second sub-theme ‘Acceptability of threat’ highlights participants’ perceptions of threat; revealing patterns of acceptance and desensitisation. Some participants discussed managing threat by ‘recognising that that’s part of the job’ (39ED: Male, RMN). Suggesting they perceive the need to accept threat as an inherent risk of providing frontline care within a low–medium secure environment. Acceptance of threat can be suggested to serve a positive purpose whereby individuals are suggested to be better able to attend to a challenge after accepting the reality of it (Shin et al., 2014). It could be argued that this is a further sign of resilience in some individuals but, the extent of acceptance and how normalised participants perceived this to be is concerning; ‘it’s just a bit scary that that can happen, but it is everyday sort of life’ (38RH: Female, HCA):

at Tesco … someone was yelling at me cos I wasn’t parking my car properly … didn’t realise they were talking to me because they weren’t particularly shouting … it’s just so low level whereas here we get called everything and spat at and all sorts kicked and punched … it’s just very blasé. 79RS (Female, HCA)

This shows participants demonstrating they have learnt to accept threat as a normalised concept and although this may mean individuals are more resilient to threat as a stressor it is concerning the potential longer term negative psychological consequences this may create if employees are not dealing with the threat using health coping strategies. Perceiving threat as ‘blasé’ suggests they are not only accepting but are also desensitised to the seriousness of threat. Furthermore, their acceptance of threat has emerged from within the workplace, into their personal lives. This is alarming as accepting threats outside of work could lead to serious endangerment or abuse for individuals. Despite the threat being a sometimes-unavoidable aspect when providing human services, it is important to acknowledge the detrimental effect threat can have on individuals (Pich, Hazelton, Sundin, & Kable, 2010). Therefore, although acceptance is perceived to help individuals cope with threat when providing frontline care, the seriousness of this should not be ignored or minimised by employees or organisations.

### Need for support

The second theme ‘Need for support’ accommodates participants experiences and perceptions of available support within a low–medium secure hospital; reflecting both the aspects central to participants feelings of being supported, as well as tensions between perceived and actual organisational support.

#### Importance of talking

The sub-theme revealed several participants identified talking to others as the main, most frequently used coping mechanisms at work:

I normally stick to making sure I’m constantly talking and if I’m sort of stressed throughout the day I’ll always go and talk to someone … being able to vent is quite important. 38RH (Female, HCA)

This trend highlighted participant’s perceived importance of talking and the ‘need to kind of rant about something if somethings just happened, we just humans … we need to find someone to listen to you really and that helps’ (92ZB: Female, RMN) when managing work-related stressors. Participants discussed benefits of being understood by ‘someone who knows exactly what happened and like talk about how rough it was’ (34WC: Female, HCA) and experiences of relief as it is difficult ‘when it all just builds up … have a bit of a rant … get it off your chest and it’s fine’ (69BN: Female, HCA). This suggests providing frontline care within a low–medium secure hospital is psychologically burdening, creating a need to offload. Moreover, perceived benefits of talking to others who can relate highlights the specific importance of peer support in helping employees continue with work. Consequently, talking appears to help workers ‘bounce back’ from work-related stressors and maintain some resiliency (Bonanno, 2004). These findings are in-line with research suggesting social sharing of stressful or emotional events can promote benefits of feeling understood, emotional relief, and helping individuals think rationally (Zech & Rimé, 2005).

#### Organisation vs. the individual

The sub-theme ‘Organisation vs the individual’ encompasses participants’ experiences and perceptions of support at work. Patterns varied depending on which type of support was discussed: individual support is characterised by colleague support (clinical staff, working at similar skill-levels) and organisation support refers to senior management support (including administrator and non-clinical staff). Participants predominantly discussed availability and engagement with colleague support: ‘it is very tense, but I think once you get used to it it’s more than anything it’s support between you and your colleagues’ (00FM: Male, HCA); expressing positive experiences and perceived benefits of this: ‘you’re in an environment where there’s lots of support from your colleagues … so you feel safe in that respect’ (39ED: Male, RMN). This suggests participants frequently and successfully seek support from colleagues when providing frontline care within a low–medium secure hospital. This trend is positive; whereby co-worker support is suggested to predict lower levels of stress and burnout amongst other human service employees (Gould et al., 2013). More specifically, co-worker support is suggested to help individuals better contend with workplace stressors; here, this is perceived to create feelings of safety among a threatening environment (Lambert et al., 2007). Additionally, this is suggested to be positively associated with staff retention (Ducharme et al., 2007) indicating positive perceptions of co-worker support may have several benefits amongst the given population.

Alternatively, participant’s experiences and perceptions of organisational support are somewhat mixed. A couple demonstrated positive perceptions of organisational support; ‘I think we’re quite supported very I mean I literally today and I went in my managers office like I’ve got a problem and he bless him left all his work and went what’s the problem’ (79RS: Female, HCA). Whereas others expressed negative perceptions; contrasting organisational support with colleague support; ‘not very supported here obviously by colleagues … but senior management not supported at all’ (69BN: Female, HCA). This suggests there are inconsistencies in how supported individuals feel via organisational support. What is concerning is negative perceptions of organisational support appear more prominent amongst data:

I think there’s a general feeling that they don’t really care about the staff … in the hospital really not particularly not individually … there’s a bit of a lip service paid to it urm we’re very very short staffed … you sometimes feel a bit not cared for. 39ED (Male, RMN)

This suggests some employees are lacking experiences of feeling personally valued, respected, or acknowledged at work; concepts which characterise low levels of workplace belongingness (Cockshaw & Shochet, 2010). This is alarming whereby perceived low levels of support are suggested to predict heightened stress and burnout (Shakespeare-Finch & Daley, 2017). Moreover, where participants expressed positive experiences of organisational support it was seen as unexpected ‘I was quite surprised and I was like taken back that they actually listened to little old me rather than say well you’re an HCA you don’t matter they actually listened’ (79RS: Female, HCA). Thus, suggesting participants experience apprehension to seek organisational support due to their lower-skilled position, feeling privileged when receiving this. This is concerning as organisational support should instead be perceived as an easily accessible aspect of any job; whereby organisations are responsible for promoting employee’s welfare regardless of position (Sparks, Faragher, & Cooper, 2001).

Several participants provided suggestions for improving organisational support; a need for ‘more acknowledgement … bit more thanks really’ (34WC: Female, HCA):

I think maybe a lot more input from management I do feel sometimes that non-clinical staff don’t quite realise how challenging and dangerous it can be … again I think just input from top management a bit more sort of everyone communicating with each other on a more frequent basis. 38RH (Female, HCA)

at least make sure it's sort of relevant to the person … I got hit about the head a little bit it wasn’t major but … I got a letter from head office here which actually was obviously just a standard printed off letter and I felt I felt it quite an insult really … a box ticking exercise. 39ED (Male, RMN)

Thus, suggesting a perceived need for organisations to demonstrate better understanding and appreciation towards frontline care workers and provide more openly offered, personalised support. Overall, this theme highlights the perceived necessity of support (especially talking) when providing frontline care within a low–medium secure hospital, but highlights inconsistencies in experiences and perceptions of available support suggesting a need for organisational change.

### Unique environment

The final theme ‘Unique environment’ captures participants’ experiences and perceptions of unique challenges and incentives when providing frontline care within a low–medium secure hospital: ‘do research before you commit to something like this cos it isn’t just an everyday job’ (38RH: Female, HCA).

#### Environmental challenges

Several participants discussed working within ‘a high stressed environment it can be very daunting … ’ whereby ‘ … the job itself is incredibly stressful’ (00FM: Male, HCA). Patterns regarding why they perceived their job/environment as stressful were identified; some challenges reflected those evident in human service occupations generally, others appear more unique to the presented sample. General challenges were: the busy nature ‘it can be quite difficult cos obviously you need to be in a thousand places at once’ (33RW: Female, HCA); staff shortages and unobtainable workloads ‘stressful … not having enough staff to facilitate the patient’s needs … we spend a lot of time in the offices as well doing paperwork when we should be with patients’ (69BN: Female, HCA); inconsistent staffing/staff-turnover:

it’s very unfair on not only the patient but also the staff I think quite often you know I’ve I’ve come in today and half my colleagues today I’ve never met before in my life and I might never see again in my life … I wish It was more of a case of having more permanent staff. 00FM (Male, HCA)

Such challenges and consequent experiences of stress are concerning, as seen in other human service occupations resulting in negative ramifications for employees, organisations, and patients (Griffin et al., 2010; Lambert et al., 2009). These issues are reflective of broader environmental issues apparent within service work whereby financial cuts and under resourcing is commonplace (Griffin et al., 2010).

Participants discussed the length of shifts (12.5 h) where despite stating they ‘prefer doing longer days’ (92ZB: Female, RMN), individuals also expressed negative experiences of this: ‘it’s a rough job like it’s 12 h shifts you get tired you get a bit burnt out … you just kind of get engulfed by it’ (34WC: Female, HCA). This suggests participants prefer the convenience of long shifts but are becoming fatigued. This is alarming whereby fatigue and burnout are suggested to compromise work performance; suggesting that the length of shift could impinge on standards of care provided to patients (Stewart & Terry, 2014). More unique challenges were discussed in relation to the type of environment participants worked in:

it’s really difficult … we’re not prison and we’re not a hospital … it’s all sort of nowhere in between … sometimes I think I’m a healthcare assistant in a hospital … other times you could be restraining so it’s like you’re in prison. 79RS (Female, HCA)

This suggests participants perceive their working environment as challenging due to being tasked with the unique challenge of fulfilling differing roles, consisting of different types of responsibility. Such experiences are in-line with research suggesting employees working within secure/correctional settings experience role conflict, feeling torn between roles of care and correction (Armstrong & Griffin, 2004). Role conflict has been shown to increase levels of workplace stress (Gallavan & Newman, 2013). Therefore, suggesting participants perceived difficulty of having to fulfil conflicting roles of care and correction is adding to workplace stress and risk of burnout within this specific population. Participants further highlighted the unique challenges of working within a ‘forensic’ environment:

it can be quite hard especially in a forensic environment like this where you can’t actually have your phones and you can’t really speak to the outside world … you are prisoned as well which is quite sad and hard. 38RH (Female, HCA)

This suggests some employees find the security requirements of a low–medium secure environment challenging, whereby they discussed feeling isolated and perceptions of imprisonment similar to patients. This is in-line with research suggesting increased security levels of similar facilities are predictive of workplace stress (Armstrong & Griffin, 2004). Finally, participants discussed the ‘unknown’ as a unique challenge: ‘an alarm goes off and we as part of a response team you go to that situation … you don’t know what you’re going into’ (79RS: Female, HCA) (often referring to patient behaviour). Participants perceived the unknown as ‘very stressful … you don’t really know what situations you’re gonna occur’ (38RH: Female, HCA), ‘medium secure is definitely quite scary’ (69BN: Female, HCA) but also interestingly as ‘part of the attraction of it as well’ (39ED: Male, RMN). This suggests they perceive their working environment as unpredictable, initiating a mix of positive and negative emotions.

#### Making a difference

In relation to the final sub-theme, despite perceiving their working environment as stressful/challenging, several participants expressed how ‘it’s not as bad as people think’ (79RS: Female, HCA); whereby their working environment can also characterise a ‘really good atmosphere’ (69BN: Female, HCA). The sub-theme ‘Making a difference’ captures positive aspects of providing frontline care within a low–medium secure hospital whereby participants interestingly discussed positive perceptions or experiences of improving patients’ situations: ‘I can go home and feel like an overwhelming sense of positivity … that you can actually make someone’s life that little bit better’ (38RH: Female, HCA). When talking about what motivates them in their job, they commonly discussed making a difference ‘I love to make a difference’ (38RH: Female, HCA). Participants expressed experiencing positive emotions due to this; providing ‘an overwhelming sense of positivity’ (38RH: Female, HCA). They believed ‘seeing how well they’ve (patients) done that makes your job worth it … we are doing something positive for these people’ (33RW: Female, HCA) and ‘thrives you to come in and do again the next day’ (38RH: Female, HCA). This suggests witnessing positive patient progression due to personal efforts provides participants with a sense of accomplishment; supporting research suggesting individuals experience satisfaction when providing successful care to others (Koen et al., 2011). Furthermore, intrinsic reward due to helping others is suggested to predict psychological well-being and lower levels of stress (Armstrong & Griffin, 2004; Pillay, 2009). Moreover, the fact participants discussed ‘making a difference’ as the main motivator of doing their job, suggests this may successfully contribute toward staff retention. Therefore, despite this overall research suggesting several factors negatively impact participant’s perceptions and experiences, it is important to emphasise the positives whereby influential aspects such as ‘making a difference’ motivate employees to continue in this occupation.

## Implications

This study qualitatively explored UK frontline care workers’ experiences and perceptions about providing care within a low–medium secure hospital. Results meet objectives, providing in-depth accounts of what delivering frontline care is like and insight into employee’s needs, offering information relevant for future practice. Key findings are presented by three main themes: Resilience to threat; Need for support; and Unique environment. Participants perceived this job as incredibly demanding and stressful. Among reasons for this were environmental challenges including staffing, workload, shift length, role conflict, security levels, and the ‘unknown’. Participants identified daily occurrences of threat but there were differences in coping strategy and resilience. The responses demonstrate negative implications, variations in coping, and desensitisation towards the seriousness of this issue. Furthermore, results concerning workplace support were mixed, whereby participants expressed predominately negative experiences and perceived a need for improvement in organisational support but discussed benefits of peer support (especially talking about work-related issues). Interestingly, despite several factors negatively effecting experiences, participants identified ‘making a difference’ as a positive motivator; making the job worth it and indicating this as a core reason for staff retention and one possible explanation for how some employees are more resilient to the stressors experienced in the workplace. Interestingly participants’ themselves did not really refer to resilience per se, indicating it is not a concept that naturally came to the mind of the individuals themselves (the evidence, or lack of evidence, for resilient behaviour, coming from the interpretation by researchers). This is not perhaps surprising as resilience was not directly raised, instead researchers wanting to get as naturalistic an insight from participants as possible.

Results add to limited understandings of the pressures faced by lower-skilled populations within human service work (Armstrong & Griffin, 2004). Findings show this population experience similar pressures as higher-skilled employees within similar fields; suggesting these occur irrespective of occupational position. Some challenges (e.g. staffing and workload) increase the understanding of general issues amongst human service work (Pillay, 2009; Shakespeare-Finch & Daley, 2017), with certain stressors (e.g. security levels and role conflict) increasing insight into unique challenges within secure facilities (Gallavan & Newman, 2013; Griffin et al., 2010). Results highlight concerning implications of identified pressures, including experiences of psychological distress, staff absenteeism, and possible turnover. This supports literature highlighting negative implications of workplace stressors on employee’s well-being and abilities to cope (Berg et al., 2006; Khamisa et al., 2015). Worryingly, findings do suggest employees may be lacking adequate support to effectively manage inherent challenges. Recent guidelines (NICE, 2018) have recommended the removal of critical incident stress debriefings for prevention of PTSD, which is perhaps counterintuitive to what individuals within the study appear to indicate needing but does reflect the lack of evidence supporting psychologically focused debriefing in this context. It should be recognised that the organisations do provide support, e.g. monthly supervision, reflective practice sessions, but it is unclear whether this occurs consistently (being cancelled due to staff shortages) or indeed is participated in meaningfully from the individual’s perspective. It could be the case that individuals who are experiencing emotional detachment may not engage with reflective practice, either by choice or through lack of capacity. What is apparent from the data is that current provision is not meeting some needs and changes to practice, or further investigation into optimum intervention for staff, is warranted.

Some participants did express abilities to persevere despite challenging circumstances, demonstrating resilience (Bonanno, 2004) but authors would also argue that there are indications that those individuals may appear more resilient, for example to the risk of harm, but their overall well-being is still be challenged through desensitisation and avoidance coping strategies. Overall, the research highlights variations in how employees are affected by and manage workplace pressures, contributing towards understandings of individual differences in perceptions and experiences of stress (Bonanno & Burton, 2013). Something bearable for one individual, may be stressful for another. Interestingly, participants demonstrating a lesser ability to cope with workplace pressures discussed more negative perceptions of organisational support. This supports research suggesting individuals receiving lower levels of support are less able to contend with workplace stressors (Gould et al., 2013). Therefore, highlighting possible inconsistencies in organisational support, suggesting this may constitute general levels of support rather than being tailored to employee’s individual needs, the latter being more preferential.

Results from this study and existing literature suggest if employees continue to feel unsupported when facing highly challenging circumstances, this risks a detrimental effect on employee’s well-being and ability to function and perform at work. This risks wider implications, predicting harmful reductions in organisational resources and overall care provided to patients; in quantity and quality (Stewart & Terry, 2014). This is alarming during a period where organisations are already experiencing financial cuts, causing resource and staff shortages (Lambert et al., 2005) and must only have been exacerbated by the additional demands created by the COVID-19 pandemic. Preliminary research indicates the challenge of healthcare service provision with increased levels of anxiety amongst healthcare service providers (Bostan, Akbolat, Kaya, Ozata, & Gunes, 2020) and this is without the additional restrictions apparent in secure facilities, for example, restrictions in visiting rights for patients. Further concerns have been apparent about the increased risk of infection within correctional facilities creating additional stress and workplace demands (Robinson, Heyman-Kantor, & Angelotta, 2020). This is a fast-moving landscape and research that has been published is often not specific to secure correctional facilities or across different countries, but a recent scoping review did evidence the extent that frontline care workers physical and mental health was been negatively impacted (Shaukat, Ali, & Razzak, 2020). Research restrictions during the pandemic mean that authors can only speculate on how workplace demands and employee stress has increased over recent months for the participants or secure hospital that was used within this study. It will be interesting to see the extent the experience of providing human service care during the pandemic heightens still further the problem of staff retention.

Taking together this highlights an integral need for organisational change and workplace intervention to improve psychological well-being and resilience amongst frontline care workers; to ensure staff retention and future ‘best’ practice.

### Strengths and limitations

This study has strengths and limitations concerning Yardley’s (2000) evaluative criteria which was adopted to ensure understanding of what constitutes high-quality research. Sensitivity to context was adhered via provision of ethical participant care whereby all participant accounts were sensitively listened to and acknowledged; referral producers were implemented to address any psychological distress. Regarding addressing relevant literature, several workplace stressors are identified amongst human service work. Consequently, although some are discussed in relation to the current results, certain precursors of stress may have offered further insight into differences in participants’ accounts, such as length of experience. Future research may wish to consider this. Further, although the research does not aim to generalise, it does still aim to have relevance to future practice. The sample chosen was purposive and has been clearly described so that interpretations offered can be situated within the context that the data was generated (Carminati, 2018; Vasileiou et al., 2018). Commitment and rigour are apparent through the systematic approach adopted to the data collection and analysis with the utilisation of multiple researchers and opportunities taken to check interpretation remains grounded in the data. A limitation, however, is that verifying the proposed interpretation with participants themselves would have provided further validity and although not an option made available to the research team it would be a preferred option in future research. To remain rigorous in approach, analysis closely followed validated stages of thematic analysis (the research team discussed and revised each stage) ensuring all relevant codes and themes were acknowledged and accurately reported. Transparency and coherence are demonstrated through detailed reports of recruitment, data collection, and analysis, with supporting evidence to ensure clarity. Lastly, impact and importance are difficult to establish directly at this stage, however, the data provides important insight into the needs of the given population which highlights implications and suggestions for future practice; with an opportunity to inform workplace interventions to improve employee well-being and resilience. Findings from this study are contributing to work with the employing organisation with an aim to address some core weaknesses in current organisation support and structure, any longer term beneficial impact is however unknown, and wider acknowledgement of a need to change is yet to be established.

### Future direction

This study is one of few to explore the experiences of a lower-skilled human service population. This, specifically within a low–medium secure environment; therefore, future research should explore whether current results are reflective of lower-skilled populations working within other types of environments. Furthermore, concerning future direction, implications of data highlight a need to develop workplace interventions to help frontline care workers better cope with workplace pressures within secure environments. The current results can be used to inform intervention. More specifically, results highlight a need for improvements in organisational support to address issues of employees feeling unsupported or desensitised towards the seriousness of pressures (for example, exposure to aggression). Interventions that draw on the positive aspects discussed by participants regarding peer support, talking to others, and the reward of ‘making a difference’ within work roles, may be more effective. Regarding feasibility, amongst a time where funding and resources are lacking, it is essential interventions are designed to ensure low-cost and easy implementation while remaining effective (Griffin et al., 2010). To effectively improve employee’s response to workplace pressures, it is recommended well-established behaviour change approaches are adopted to inform the design and evaluation of interventions (Michie & West, 2013). The COM-B model constitutes a simple behaviour change approach, incorporating individual’s capability, opportunity, and motivation for behaviour change (Michie & West, 2013). This, or similar approaches, may help address unhelpful barriers or individual differences preventing employees from effectively coping at work. Consequently, interventions informed by the current data may improve resilience amongst frontline care workers, ensuring employee ‘best’ practice within low–medium secure environments.

## Conclusion

Overall, this study provides valuable insight into frontline care workers’ experiences of working within a low–medium secure environment. Results highlight implications of workplace pressures on employee well-being highlighting needs for organisational change. In-depth understanding of employee’s needs offer opportunity to inform the design of workplace interventions to improve employee resilience and psychological well-being. Consequently, if successful, interventions could improve employee’s abilities to effectively cope at work, improving satisfaction, and staff retention. Accordingly, this may improve current issues within human service work concerning employee stress and burnout, organisational resources, and the quality of care provided to vulnerable individuals.

## Supplementary Material

Supplemental Material
