# Supplementary material for: ‘Don’t show that you’re scared’: resilience in providing healthcare in a UK low-to-medium secure hospital
Source: Health Psychol Behav Med. 2021 Jan 28;9(1):84–103. doi: 10.1080/21642850.2021.1874956 (PMC8158232; doi:10.1080/21642850.2021.1874956)
Supplement: Supplemental Material [file RHPB_A_1874956_SM8977.docx]

**Appendix A**

INTERVIEW GUIDE

**Providing frontline care in a low to medium secure hospital – a healthcare workers perspective.**

*Use the guide to cover core topic areas, suggestions for prompts are provided*

**The Job**

- Firstly, could you please just tell me a bit about the nature of your work?
- What are your main responsibilities?
- What does a ‘normal’ day at work entail?
- What do you believe is expected of you?
- Why did you choose to do your job?
- How/why did you enter your field of work?
- What motivates you to do your job?
- What do you believe to be beneficial about your job?
- Why do you choose to stay in this job?

**The Environment**

- How would you describe your working environment?
- How does it compare with other environments?
- What is it like working within a low-medium secure environment?
- How does this affect: you personally? /others? /your work?
- Do you have any concerns about working within a ‘secure’ environment?

**Patient Interactions**

- Within your work environment, what sorts of interactions do you have with patients?
- What sorts of relationships do you have?
- How do these interactions make you feel?
- Based on personal experiences, what is it like providing care to patients?
- How do you find this task?
- How do you ensure you are prepared? (personal preparation/ training/ support)
- Is there anything that is challenging?
- How does this affect you? /How do you cope with this?

**Challenging Situations**

- Within your field of work, how/what would you describe to be a ‘daily challenge’?
- Is there a particular instance you can think of that you have found challenging?
- How did you manage/ cope with this?
- How do you ensure you are prepared for these situations?
- What effect do you believe this has on you/ others/ the organization?

Can you think of an example of a particularly challenging situation that occurs within the workplace?

**Experience of violence and aggression**

- Can you think of any examples where you have felt unsafe while at work?
- What/ who made you feel unsafe?
- How did you react? / what impact did this have on you or your work?
- Have you ever experienced or witnessed any violent or aggressive behaviour while at work?
- How did this affect you (How did you respond/ feel/ cope or move past this?)
- How were you supported? (who supported you/ how helpful was this?)
- What impact did this have on your work?

**Coping and Support (Self/ Organisation/ Others)**

- What do you believe it takes to cope with your job?
- How do you feel you cope with your job?
- How does your job affect you emotionally/physically/personally?
- How do you ensure you care for your own well-being, while also caring for others?
- How do you cope emotionally?
- How do you provide yourself self-care?
- How do you keep your work separate from other areas of your life?
- Please could you describe what support you are offered/ receive at work?
- Who are you supported by? (personal, organizational, others)
- When are you supported? (regularly, dependent on event)
- How supported do you feel?
- How helpful do you find this?
- What do you think could be done to help you better cope with your work/ improve your job role?

*Thank the participant for their time and honesty.*

*Ask if they have any questions.*

*Debrief*
